# Analysis of the Curative Effect of Posterior Approach on Lumbar Brucellar Spondylitis with Abscess through Magnetic Resonance Imaging under Improved Watershed Algorithm

**DOI:** 10.1155/2021/1933706

**Published:** 2021-07-10

**Authors:** Zuoji Feng, Xiaomei Wang, Xiling Yin, Jingqi Han, Weijie Tang

**Affiliations:** ^1^Department of Orthopaedics, Huangdao District Hospital of Traditional Chinese Medicine, Qingdao 266500, China; ^2^Operating Room, Huangdao District Hospital of Traditional Chinese Medicine, Qingdao 266500, China; ^3^Department of Nursing, Huangdao District Hospital of Traditional Chinese Medicine, Qingdao 266500, China; ^4^Department of Imaging, Huangdao District Hospital of Traditional Chinese Medicine, Qingdao 266500, China

## Abstract

To explore the performance of improved watershed algorithm in processing magnetic resonance imaging (MRI) images and the effect of the processed images on the treatment of lumbar brucellar spondylitis (BS) with abscess by the posterior approach, the watershed algorithm was improved by adding constraints such as noise reduction and regional area attribute. 50 patients with abscessed lumbar disc herniation admitted to the hospital from January 2018 to January 2019 were selected, and all of them were examined by MRI. They were rolled into two groups in random. The treatment group (*n* = 25) accepted surgery with the aid of MRI images processed by the improved watershed algorithm, and the control group (Ctrl group) (*n* = 25) accepted surgery with the aid of unprocessed MRI images. The improved watershed algorithm can accurately segment the spine, and the segmentation results were relatively excellent. In contrast with the unprocessed MRI image, that processed by the improved watershed algorithm had a positive effect on the operation. In contrast with the Ctrl group, the visual analogue scale pain score (VAS), oxygen desaturation index (ODI), erythrocyte sedimentation rate (ESR), and high sensitivity C-reactive protein (CRP) were obviously lower (*p* < 0.05). The improved watershed algorithm proposed performs better in MRI image processing and can effectively enhance the resolution of MRI images. At the same time, the posterior approach has a good effect in the treatment of lumbar BS with abscess and is worthy of clinical promotion.

## 1. Introduction

With the rapid development of modern society, the pace of people's lives is accelerating; at the same time, more and more people have the spinal diseases. Diseases such as lumbar disc herniation, cervical spondylosis, and curvature of the spine are very common clinically, and these diseases pose a serious threat to people's normal life and work [1, 2]. Statistically, the incidence of scoliosis in adolescents in China is 20%, and 40% of adults have unhealthy spine [3]. In recent years, brucellosis has attracted more and more attraction for their pathogenicity worldwide. Body infections and complications have brought great suffering to people's lives and at the same time increased the economic burden of society. Therefore, it has become a worldwide public health problem [4]. When *Brucella* invades the vertebral body or intervertebral disc tissue, it will cause spinal infection, called brucellar spondylitis (BS). Its incidence is also increasing year by year [5]. The use of medication alone for the treatment of BS is often unsatisfactory. At the same time, there are reports showing that for patients whose drug treatment is not effective, simply prolonging the treatment time will not improve the treatment effect, but will increase the damage to the liver and kidney function [6]. On the basis of medical treatment of lumbar brucellosis spondylitis, surgical operations can directly reach the diseased vertebral body and intervertebral space, remove infected lesions, inflammatory granulation tissues, and abscesses, scrape off the sclerotic bone, and create good conditions for bone healing in the bone graft area. In short, it can effectively shorten the course of antibiotic treatment and reduce the incidence of adverse reactions. However, the results of surgical procedures are largely influenced by preoperative imaging [7]. At this stage, the pathological conditions of intervertebral discs and vertebrae are mainly displayed by MRI. MRI images contain important information about the intervertebral discs and vertebrae, which are of great significance for the diagnosis and treatment of spinal diseases [8]. At present, there are more research studies on the segmentation algorithm of the brain, heart, and spine. The algorithm for the brain and heart is nearly mature, while that for the spine still needs to be improved [9]. Presently, most commonly used intervertebral disc positioning and segmentation algorithms are grayscale features, Gbor features, and shape information. They combine the algorithm with image features and prior information. The commonly used algorithms include methods based on the deformation models, Hough transformation method, wavelet transform, and watershed algorithm. The watershed algorithm is a processing method based on morphology. Since this method can also have a good effect on weaker edges, it has become a research hotspot in recent years [10]. However, traditional methods are prone to oversegmentation; in this study, the algorithm was improved.

Therefore, in the study, the watershed algorithm was improved. Then, the effect of MRI images processed by the improved watershed algorithm in the posterior approach treatment of abscessed lumbar brucellosis was further studied.

## 2. Methods and Materials

### 2.1. Design and Improvement of Watershed Algorithm

The watershed algorithm is improved according to the following steps.

(I) First, the medical images with unclear contrast and unclear boundaries are preprocessed. In this experiment, the grayscale linear transformation is mainly used to process the image, as shown in equation (1), where *I*_*i*_ represents the input image, *I*_*o*_ represents the output image, and the *A*_1_ parameter has an important influence on the result. When it is greater than 1, the image contrast is enhanced, and when it is less than 1, the image contrast is reduced. *A* is set to 2.
  (1) $$\begin{array}{c} I_{o}=A_{1}\cdot I_{i}+A_{2}. \end{array}$$
(II) Extraction of internal markers: the internal markers represent the target, so it is particularly important to mark the disc area. The brightness of the intervertebral disc in the spine MRI image is relatively high, so the content can be extracted through binarization. In this study, the maximum between-class variance method is used to further binarize the preprocessed image, as shown in the following equation.
  (2) $$\begin{array}{c} \text{Var}\left(X\right)=\sum_{i=1}^{n}p_{i}\cdot {\left(x_{i}-\mu \right)}^{2}=\sum_{i=1}^{n}\left(p_{i}\cdot x_{i}^{2}\right)-\mu^{2}. \end{array}$$
(III) External label extraction: after the internal label is obtained, it is necessary to further mark the pixels of the background accordingly, that is, external label extraction. In this study, Euclidean distance was used to transform the internally marked image, as shown in equation (3). Euclidean distance transform can calculate the distance of the background grid points in the figure, as shown in equation (4).
  (3) D=x1−x22+y1−y22,
  (4) $$\begin{array}{c} D_{ab}=\min\left\{D\left[\left(x,y\right),\left(a,b\right)\right]|\left(a,b\right)\in M\right\}. \end{array}$$
(IV) Improvement of the existing algorithm: through the research of the above algorithm, the internal markers are extracted more accurately. The specific execution process is as follows: first, each area with a gray value of 1 in the connected area is marked and the 8-connected domain is calculated. Then, the total number of pixels in each area is counted, and the interference area is removed. Finally, the processing result is obtained.

### 2.2. Research Subjects and Grouping

50 patients who were diagnosed as abscessed lumbar keyboard herniation from the hospital from January 2018 to January 2019 were selected, and they were between 34 and 65 years old. All patients had night sweats, fatigue, loss of appetite, and varying degrees of persistent low back pain or lumbosacral pain. Some patients experienced low back muscle spasm. In severe cases, the lumbar spine motor function was restricted. As a result, they could not turn over, stand, or walk, while they were partly accompanied by scoliosis and mild kyphosis. All patients participating in the experiment signed informed consent forms, and this study had got permission from the hospital ethics committee.

The patients were rolled into the experimental group and the Ctrl group in random. The experimental group was subjected to posterior median surgery with the aid of MRI images processed by the improved watershed algorithm, and the Ctrl group was subjected to that with the aid of unprocessed MRI images.

### 2.3. Inclusion Criteria and Exclusion Criteria

All included patients were chosen according to diagnosis and treatment of brucellosis established by the Ministry of Health in the epidemiology, clinical manifestations, and experimental tests. The inclusion criteria include the following. Imaging tests showed that the lumbar spine had infectious lesions and were accompanied by vertebrae paradural or epidural swelling in the spinal canal. After a period of drug treatment, the symptoms had not improved obviously or the systemic symptoms had improved, but there was still obvious local neurological dysfunction. Only the posterior approach abscess internal fixation was performed.

Exclusion criteria include the following. Patients suffering from lumbar BS with abscess also had other infectious spondylitis. The structure of the anterior column of the vertebral body was severely damaged. There were more serious chronic diseases or infectious diseases. The patient was accompanied by cardiovascular and cerebrovascular diseases and nephropathy at the same time. Patients without regular drug treatment.

### 2.4. Surgical Treatment and Evaluation Indexes

4–6 weeks before surgery, patients took 0.1 g doxycycline once a day, with 0.6 g rifampin each time. Surgery was performed using the posterior midline approach, and the patient underwent general anesthesia. An incision was made at the posterior midpoint of the lumbar spine. A blunt separation was performed at the incision site to expose the adjacent vertebral bodies at the upper and lower adjacent positions of the lesion. Then, they were peeled along the line to the articular processes on both sides and pedicle screws were used to fix them. If the damage of the diseased vertebrae was relatively light and the bone quality of the pedicle and the nail was good, the screws with streptomycin should be placed in the diseased vertebrae. Otherwise, it needed to cross the diseased vertebrae for corresponding fixation operations. The lamina was removed for decompression. After the spinal cord, nerve roots, and cauda equina tissues were exposed, the spinal canal of inflammatory granulation or necrotic tissue around the dural and lateral saphenous fossa and below the posterior longitudinal ligament, as well as localized encapsulated pus, was cleared. If inflammatory damage or hardened bone occurred at the edge of the vertebral body, a small curette was applied to scrape. If there was a diseased fibrous ring at the incision, curettage on the diseased nucleus pulposus and necrotic inflammatory tissue was performed, and the sclerotic bone tissue was further removed. When abscesses and inflammatory tissue appeared behind and near the longitudinal ligament, a disc mirror and a multiangle curette were adopted to carefully clean it. When the joint process was damaged, the lesion was removed on the same side. A large amount of gentamicin-containing normal saline was used to wash the lesion repeatedly. Then, the posterolateral transverse process and the articular process were polished and grafted. The bone graft was mixed with 1 g streptomycin, and the drainage tube was placed, and the incision was closed. H&E staining was performed on the vertebral body tissues of the two groups of patients during and after the operation. Specifically, a slice with a thickness of 4 *μ*m was made, and the H&E staining was performed after the film was taken out and baked. The specific operations were as follows. The paraffin section was dewaxed with xylene solution, dehydrated with gradient alcohol for 5 seconds, and then washed with water. After hematoxylin staining for 5–10 minutes, the excess dye solution was washed away. Then, the sample was acidified by hydrochloric acid glycol. After that, the sample was dyed with eosin solution for 3–5 minutes to remove the excess dye solution. Subsequently, it was dehydrated and cleared with gradient ethanol for 5 seconds, and the slices were dried overnight. Finally, images were taken and observed under the microscope, and the image magnification was 200x.

After that, VAS, ODI, ESR, and CRP before surgery, 2 weeks after surgery, and 1 year after surgery were compared. The total score of VAS was 10 points. 0∼2 points indicated comfortable; 3∼4 points indicated mild discomfort; 5∼6 points indicated moderate discomfort; 7∼8 points indicated severe discomfort; 9∼10 points indicated extreme discomfort. The ODI index was divided into ten items, and the score of each item ranged from 0 to 5, with 6 levels from light to heavy. These items included low back pain and leg pain, personal living conditions, lifting weights, walking, sitting, standing, and sleeping status, sexual life status, social life status, and travel status.

### 2.5. Statistical Analysis

The SPSS 22.0 was adopted to process the data, and the mean ± standard deviation was adopted to express the counting results. The *t*-test was adopted for comparison between the two groups, and *p* < 0.05 meant the difference was obvious.

## 3. Results

### 3.1. Image Segmentation Results

**Figure 1:**
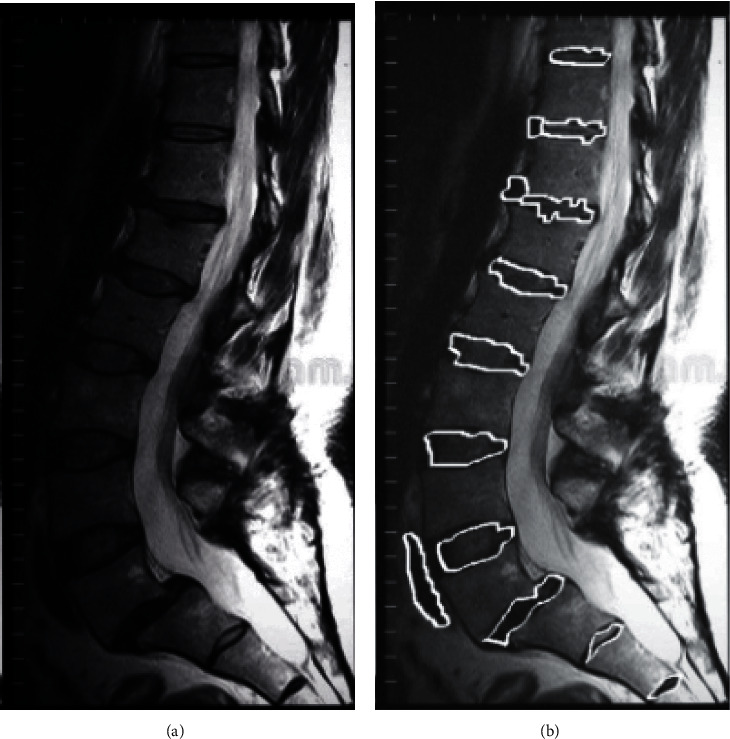
The segmentation image of improved watershed algorithm. (a) Spine image. (b) Segmentation result.

The same spine image was selected for corresponding processing, and the segmentation result is shown in Figure 1. The improved marked ridge algorithm did not have the problem of oversegmentation. At the same time, the improved marked watershed algorithm can segment the spine in a one-time, fully automatic, and unsupervised manner.

**Figure 2:**
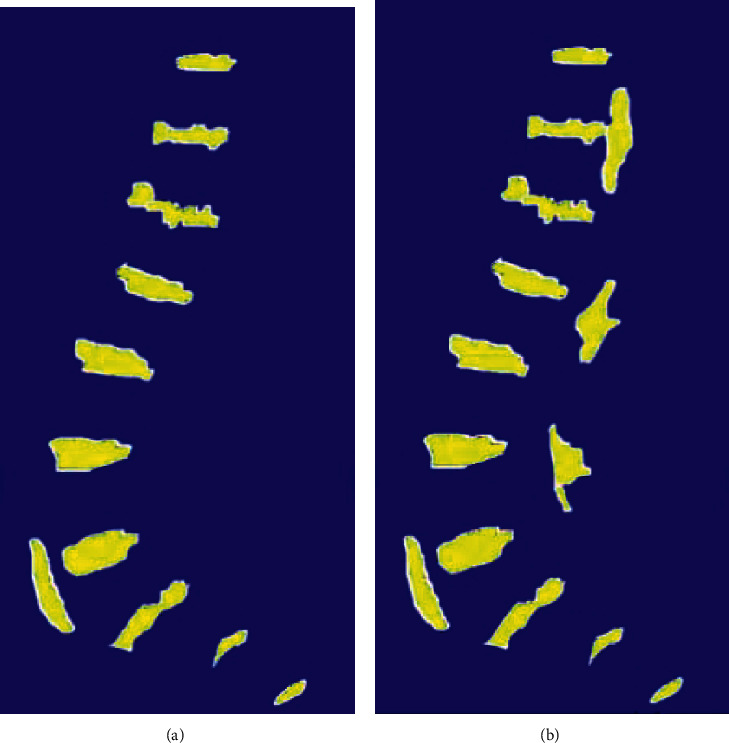
The pseudocolor images. (a) The algorithm proposed. (b) CLAHE watershed algorithm.

The algorithm proposed was further compared with the algorithm based on contrast-limited adaptive histogram equalization (CLAHE). At the same time, to make the comparison result more obvious, the image was processed as a color map, and the result is shown in Figure 2. It was evident that the contrast-limited adaptive histogram watershed was not accurate in positioning the edge of the intervertebral disc and there were many interference areas.

### 3.2. Comparison of Segmentation Accuracy

**Figure 3:**
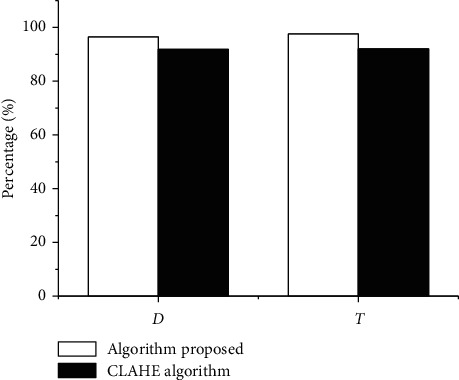
Comparison of segmentation accuracy.

The segmentation overlap ratio was expressed as *D*, and *T* represented the ratio of the target pixel segmented by the algorithm to all the correct area pixels segmented by the expert. Both values were between 0 and 1, and the result is shown in Figure 3. In contrast with the CLAHE watershed algorithm, the proposed algorithm had better segmentation performance for the intervertebral disc, and the segmentation result was more accurate.

### 3.3. Comparison of General Data

**Figure 4:**
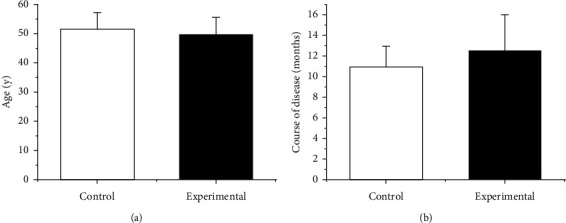
Comparison of general patient data. (a) Age. (b) Course of disease.

There were 16 males in the experimental group, accounting for 64.00%. They were 35–65 years old, with an average age of 49.53 ± 6.12 years, and the course of disease was 8–31 months, with an average of 12.45 ± 3.55 months. There were 13 males in the Ctrl group, accounting for 52.00%. They aged from 31 to 67 years, and the average age was 51.27 ± 6.11 years; the course of disease was 6–21 months, with an average of 10.85 ± 2.16 months. The general information was compared, and the results are shown in Figure 4, *p* > 0.05.

### 3.4. Comparison of Pathological Examination

**Figure 5:**
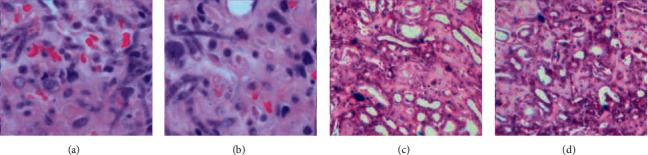
Results of H&E staining. (a) Ctrl group before surgery. (b) Experimental group before surgery. (c) Ctrl group after surgery. (d) Experimental group after surgery.

The pathological examinations were performed on the lesion tissues before and after the operation, and the results are shown in Figure 5. Before the operation, the pathological slices of the two groups showed obvious brucellosis microscopic characteristics, and *Brucella* with uniform and almost uniform morphology could be seen, which was tiny spherical, ball-rod, and oval, and inflammatory reactions were also visible. After the operation, *Brucella* in the experimental group and the Ctrl group disappeared. At the same time, the bone tissue examination results of the nail channel taken during the operation showed that there was no inflammatory reaction in both groups.

### 3.5. Comparison of Preoperative and Postoperative Pain Scores and ODI Index

**Figure 6:**
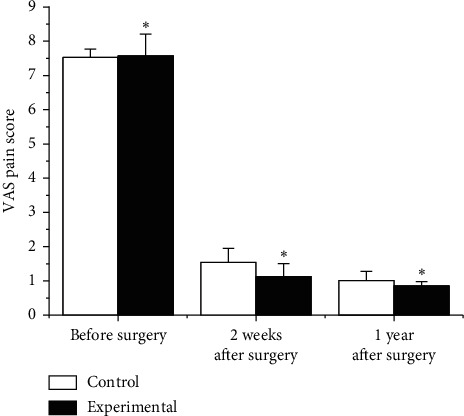
Comparison of VAS scores.

According to the VAS pain score, the preoperative and postoperative pain was compared, and the results are shown in Figure 6. Before the operation, the VAS score of the experimental group was 7.57 ± 0.64, and the VAS score of the Ctrl group was 7.53 ± 0.24 (*p* > 0.05). The VAS scores after treatment were compared. The VAS scores of the experimental group were 1.31 ± 0.38 and 0.86 ± 0.12, 2 weeks and one year after treatment, and those of the Ctrl group were 1.54 ± 0.41 and 1.01 ± 0.27. In contrast with the Ctrl group, the experimental group had obviously lower VAS scores 2 weeks after operation and 1 year after surgery, *p* < 0.05.

**Figure 7:**
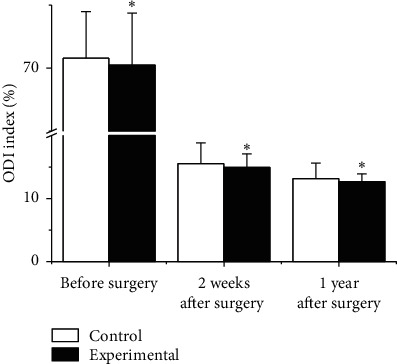
Comparison of ODI index.

According to the lumbar spine dysfunction index, the preoperative and postoperative pain was compared, and the results are shown in Figure 7. Before surgery, the ODI index (%) of the experimental group was 70.24 ± 4.11 and that of the Ctrl group was 70.78 ± 3.69 (*p* > 0.05). The ODI index (%) after treatment was compared. The ODI index (%) of the experimental group was 14.96 ± 2.14 and 12.68 ± 1.22, 2 weeks and 1 year after treatment in the experimental group, and that of the Ctrl group was 15.52 ± 3.33 and 13.17 ± 2.45, respectively. In contrast with the Ctrl group, the ODI index (%) of the experimental group 2 weeks after operation and 1 year after operation was obviously lower, *p* < 0.05.

### 3.6. Comparison of Preoperative and Postoperative ESR and CRP Scores

**Figure 8:**
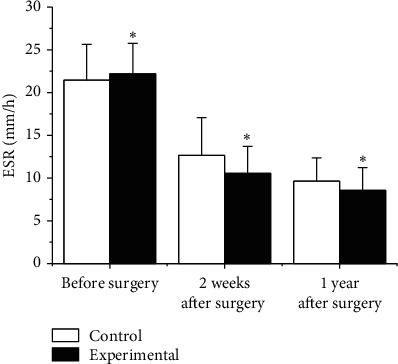
Comparison of ESR.

The preoperative and postoperative ESRs were compared, and the results are shown in Figure 8. Before surgery, the ESR of the experimental group was 22.17 ± 3.58 mm/h and that of the Ctrl group was 21.45 ± 4.18 mm/h (*p* > 0.05). The ESR after treatment was compared. The ESR of the experimental group was 10.56 ± 3.14 mm/h and 8.55 ± 2.66 mm/h, 2 weeks and 1 year after treatment, and that of the Ctrl group was 12.66 ± 4.41 mm/h and 9.63 ± 2.72 mm/h. In contrast with the Ctrl group, the ESR of the experimental group was obviously lower 2 weeks after surgery and 1 year after surgery, *p* < 0.05.

**Figure 9:**
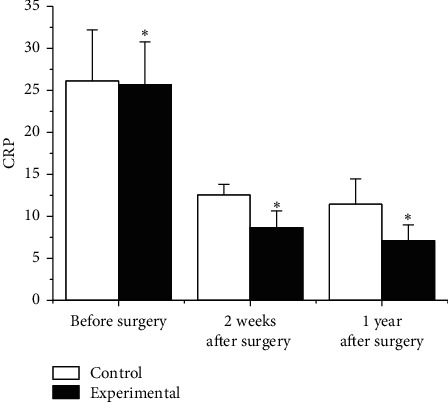
Comparison of CRP.

The preoperative and postoperative CRP was compared, and the results are shown in Figure 9. Before surgery, the CRP of the experimental group was 25.68 ± 5.11 and that of the Ctrl group was 26.14 ± 6.09, *p* > 0.05. The CRP after treatment was compared. The CRP of the experimental group was 8.66 ± 1.99 and 7.11 ± 1.87 after 2 weeks of treatment and that of the Ctrl group was 12.57 ± 1.24 and 11.45 ± 3.01. In contrast with the Ctrl group, the experimental group had obviously lower CRP at 2 weeks and 1 year after surgery, *p* < 0.05.

## 4. Discussion

Spine MRI images are based on the magnetic resonance phenomenon of hydrogen in the human body, which is important to the diagnosis of common spinal diseases such as scoliosis, disc herniation, scoliosis, and disc degeneration [11]. The research of Bozbaş et al. showed that MRI at this stage often showed blur and low contrast due to multiple factors [12]. Alyousef and Aldoghaither mentioned in the study of the current spine image segmentation algorithm that the watershed algorithm had inherent advantages due to its morphological characteristics. Therefore, the watershed algorithm was further improved on this basis [13]. In this study, patients with lumbar BS with abscess were treated with a posterior approach, and relatively good results were obtained, similar to the results of Xiong et al. that the posterior approach was more effective in treating abscessed lumbar spine brucellosis spondylitis than the cross-image approach [14]. At present, there are many surgical methods for the treatment of abscessed lumbar brucellar spondylitis. In the past, anterior lesion removal combined with posterior internal fixation was commonly used. The advantage is that it can fully expose and completely remove the lesion and surrounding abscesses under direct vision, especially paravertebral or huge abscess of the psoas. At the same time, it avoids the direct contact between the fixture and the lesion and reduces postoperative infection and recurrence. In recent years, with the development of spine surgery techniques, some scholars have gradually begun to try to treat lumbar brucellosis with abscesses only by the posterior approach. Research on the treatment of infectious spinal lesions suggested that while the lesion was removed, restoring or rebuilding spinal stability was an important prognostic factor [15–17]. Enjin et al. have shown that posterior pedicle internal fixation can provide at least 60% and 80% longitudinal and axial holding forces, while anterior cancellous bone fixation can only provide 15–20% power of control [18]. In the study, it was found that the VAS score and ODI index of the both groups dropped obviously after surgery, and the experimental group was slightly better than the Ctrl group. It indicated that the posterior approach can effectively improve the pain and clinical symptoms. At the same time, the processed MRI image had certain guiding significance for surgery. The application of MRI images is relatively mature at this stage, but the accuracy of diagnosis is often affected by the quality of the images. Unuvar et al. also stated in the study that poor image quality would largely affect the judgment of doctors [19].

## 5. Conclusion

In the study, the watershed algorithm was improved and verified. It was found that the improved algorithm can segment and optimize the spine image well. Then, the effect of MRI images processed by the improved watershed algorithm on the posterior approach treatment of lumbar BS with abscess was further studied. It was found that in contrast with the Ctrl group, the experimental group had lower VAS, ODI index, ESR, and CRP at 2 weeks and 1 year after surgery (*p* < 0.05). It can be inferred that for lumbar BS patients with paraspinal or intraspinal abscess displayed by MRI imaging, if their local symptoms or neurological symptoms do not improve significantly after standardized drug treatment, reasonable choice of simple posterior lesion removal and internal fixation can also achieve satisfactory clinical results. However, the accuracy of the algorithm improved is limited by the contrast of the image. At the same time, the sample of this experiment is small and the type of patient's disease has not been subdivided, which needs further exploration.

## Data Availability

No data were used to support this study.
